# Diagnostic and prognostic potential of the oral and gut microbiome for lung adenocarcinoma

**DOI:** 10.1002/ctm2.508

**Published:** 2021-09-26

**Authors:** Mi Young Lim, Seungpyo Hong, Kum Hui Hwang, Eun Jin Lim, Ji‐Youn Han, Young‐Do Nam

**Affiliations:** ^1^ Research Group of Healthcare Korea Food Research Institute Wanju‐gun Republic of Korea; ^2^ Center for Lung Cancer National Cancer Center Goyang‐si Republic of Korea

Dear Editor,

Lung cancer is one of the most common cancers.^1^ Although its primary risk factor is smoking,^2^ 15–20% and >50% of male and female lung cancer patients, respectively, are nonsmokers,^3^, ^4^ which indicates the involvement of other risk factors. Recently, alteration of the oral and gut microbiomes was observed in lung cancer patients,^5^, ^6^ suggesting potential roles of microbiota in lung cancer. We investigated the oral and gut microbiomes of never‐smoking lung adenocarcinoma patients to identify microbial signatures of lung cancer, which can be used for diagnosis and prognosis.

Oral and gut microbiomes were profiled from never‐smoking lung adenocarcinoma patients (cancer, *n* = 91) and from age‐ and sex‐matched never‐smoking healthy controls (control, *n* = 91). Most patients were female (*n* = 84, 92.3%) and had stage IV lung cancer (*n* = 81, 89.0%; Table S1). Shannon's diversity index of the oral microbiome was significantly lower in patients, and the overall oral microbiome structure differed significantly between patients and controls (Figure 1A). The gut microbiome structure also differed between patients and controls, but the difference in Shannon diversity was insignificant (Figure S1A).

**FIGURE 1:**
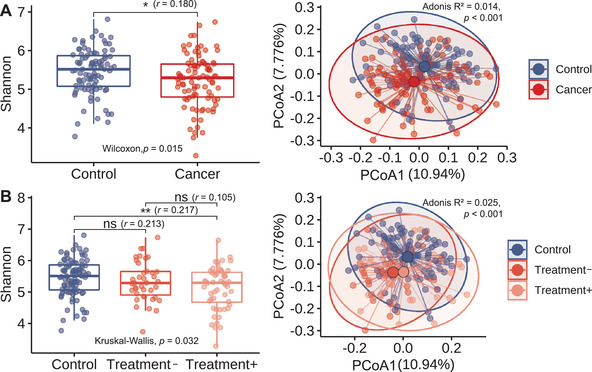
Oral microbiome diversity in healthy controls and lung cancer patients. Shannon's diversity index (left) and principal coordinate analysis plots (right) based on the Bray–Curtis distance. Samples were divided into (A) healthy controls and lung cancer patient groups, or (B) healthy controls, Treatment–, and Treatment+ groups. *p < 0.05; **p < 0.01; ns, not significant; Wilcoxon rank‐sum test, *r* means Wilcoxon effect size

The patients were further categorized into treated (Treatment+, *n* = 52) and untreated groups (Treatment–, *n* = 39) based on, at the time of microbiome sampling, whether they had received one or more lines of chemotherapy or targeted drug therapy for lung cancer since their first lung cancer diagnosis. The oral microbiome diversity was reduced in the Treatment– group (Wilcoxon rank‐sum, *p* = 0.21) and reduced more in the Treatment+ group (Wilcoxon rank‐sum, *p* = 0.01; Figure 1B). No significant difference in the gut microbiome diversity was observed irrespective of treatment status (Figure S1B). Regardless of the oral and gut microbiomes, the samples in the Treatment– and Treatment+ groups were located closer to each other on the principal coordinate analysis plot than to the control samples (Figure 1B and Figure S1B).

We further compared the relative abundance of genera in the oral microbiome of the patients and controls. *Veillonella* was more abundant in the patients, whereas the abundance of 15 genera—*Mogibacterium*, *Butyrivibrio*, *Variovorax, Ralstonia, Catonella, Bulleidia*, and *Oribacterium*—decreased in the patients (Wilcoxon rank‐sum test, false discovery rate [FDR] < 0.1; Figure 2A). These seven genera were significantly less abundant in the patients regardless of treatment history (Figure 2B,C). By contrast, some taxa were associated with the treatment condition: *Olsenella* was more abundant in the Treatment+ patients, whereas *Veillonella* was more abundant in the Treatment– patients (Figure 2B,C).

**FIGURE 2:**
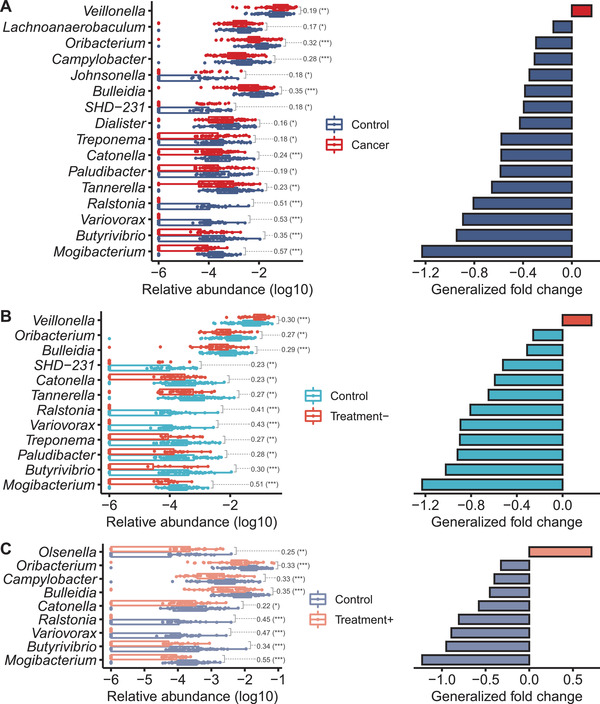
Oral bacteria genera that were differentially represented in the lung cancer patients and control groups. Log_10_‐transformed relative abundance (left) and generalized fold change (right) of differentially abundant genera among the different groups of subjects. Differentially abundant genera in healthy controls versus (A) all lung cancer patients, (B) Treatment– group, or (C) Treatment+ group were identified using a Wilcoxon rank‐sum test (*q*  <  0.1). *q < 0.1; **q < 0.05; ***q < 0.01; the numbers between groups in the boxplot indicate the Wilcoxon effect size

Compared with oral bacteria, a relatively small number of gut bacterial taxa was associated with lung cancer. *Clostridium*, *Enterococcus*, and *Streptococcus* were enriched in patients, whereas *Faecalibacterium* was enriched in controls (Wilcoxon rank‐sum test, FDR < 0.1; Figure S2A). A similar difference was observed between the Treatment+ and control groups, but not between the Treatment– and control groups (Figure S2B). Considering that microbes in the oral cavity can potentially migrate to the lungs,^7^ they may be more strongly associated with lung cancer than gut microbes.

Although the functions of lung cancer–associated microbes are not clearly understood, there are some references about them. *Veillonella* can activate cancer signaling pathways in airway epithelial cells,^8^ and the enrichment of *Enterococcus* and *Streptococcus* in the gut microbiome is linked to colorectal cancer.^9^ *Faecalibacterium* can modulate systemic immune responses.^10^ We further discussed the functional implications of these microbes in the Supporting Information.

We then evaluated whether the cancer status can be predicted from oral microbiome profiles. The patients were successfully distinguished from the controls using a machine learning model, with an area under the receiver operating characteristic curve (AUC) of 0.95 in the fivefold cross‐validation tests (Figure 3A and Figure S3). We further confirmed the performance of predictive models on an independent dataset including 41 patients with nonsmall cell lung carcinoma and 612 healthy controls (AUC = 0.88). The predictive model trained with the control and Treatment+ groups could distinguish cancer status (AUC = 0.96) better than the model trained with the control and Treatment– groups (AUC = 0.88; Figure 3A). The predictive performance on the independent dataset was also higher for the model trained with the Treatment+ group (AUC = 0.86) than that with the Treatment– group (AUC = 0.80). Although the prediction performance slightly varied, a similar set of microbial taxa was used in the two models (Figure 3B). Hence, the oral microbial taxa identified herein represent a general signature of lung cancer and can be used as diagnostic biomarkers.

**FIGURE 3:**
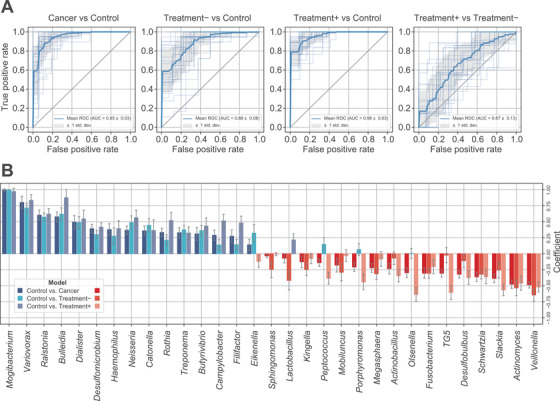
Lung cancer prediction from oral microbiome signatures. (A) Receiver operating characteristic (ROC) curve of the predictive models that distinguish the total cancer patients, Treatment+ group, Treatment– group, and control group from each other. The ROC curves of the test sets are plotted with semi‐transparent lines, and the average curve is plotted with a solid line. The area representing one standard deviation from the average is colored in gray. The area under curve (AUC) is shown in the islet. (B) Normalized coefficients of the predictive models (Bayesian ridge). The genera for which the coefficients belonged to the highest and lowest 5th percentiles in the predictive model of cancer versus control are displayed. The higher abundance of a microbe with a positive coefficient increases the chance of predicting a healthy control

Cancer status could also be predicted using gut microbiome profiles, but its predictive power was limited (AUC = 0.76; Figure S4A); the performance on the independent dataset was lower (AUC = 0.67). The gut microbiome of the Treatment+ group was slightly more informative in predicting the cancer status than that of the Treatment– group (Figure S4A). Similar genera were used in both models (Figure S4B), and their performances on the independent dataset were similar (AUC = 0.67, for both models, respectively).

To evaluate the prognostic impact of microbiome profiles, we clustered the patients into two groups by their oral microbiome similarity (Figure 4A). For the overall microbial structure, the first group (Cluster 1) was more dissimilar from the controls than the second group (Cluster 2; Figure 4B). In the Treatment– group, although statistical significance was not achieved, Cluster 2 patients had a trend toward longer survival than Cluster 1 patients (*p* = 0.14; Figure 4C). *Streptococcus* and *Megasphaera* were more abundant and *Haemophilus* was less abundant in the group with a worse survival outcome (Cluster 1; Figure 4D,E). However, no significant difference was observed in survival outcome among the Treatment+ patients.

**FIGURE 4:**
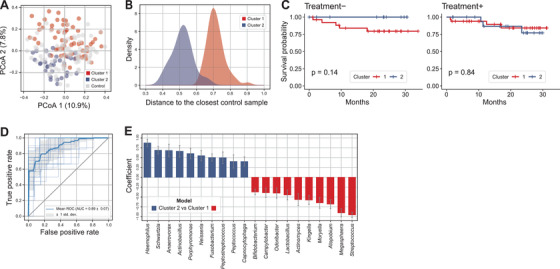
Prognostic impact of the oral microbiome profiles for lung cancer patients. (A) Principal coordinate analysis plot of the oral microbiome. Lung cancer patients were clustered into two groups based on Bray–Curtis distances. (B) Density plot of oral microbiome clusters based on the distance to the control sample with the highest similarity. (C) Survival curves for the two clusters of oral microbiome profiles. (D) Receiver operating characteristic curve of the predictive models that distinguish Clusters 1 and 2. (E) Normalized coefficients of the predictive models (Bayesian ridge)

Collectively, we found the alterations in the oral and gut microbiomes associated with lung adenocarcinoma and cancer treatment. The predictive models based on oral microbiome profiles could successfully distinguish the lung cancer patients from the healthy controls. These findings suggest the possibility of diagnosis of lung cancer, especially for nonsmokers, using oral microbiome profiles.

## CONFLICT OF INTEREST

The authors declare no conflict of interest.

### ETHICS APPROVAL

This study was performed with approval from the National Cancer Center Institutional Review Board (approval number NCC2016‐0208). All the participants provided written informed consent.

### DATA AVAILABILITY STATEMENT

The oral and gut microbiome data of never‐smoking patients with lung adenocarcinoma are available in the European Nucleotide Archive (https://www.ebi.ac.uk/ena/) under accession numbers PRJEB44168 and PRJEB44169, respectively.

### AUTHOR CONTRIBUTIONS

Ji‐Youn Han and Young‐Do Nam conceived and designed the study. Kum Hui Hwang, Eun Jin Lim, and Ji‐Youn Han collected the patients’ samples and provided the clinical information. Mi Young Lim performed the experiments. Mi Young Lim and Seungpyo Hong analyzed the data and wrote the manuscript. All authors reviewed the manuscript. The authors read and approved the final manuscript.

## Supporting information



Supporting Information
