# Supplementary material for: Diagnostic and prognostic potential of the oral and gut microbiome for lung adenocarcinoma
Source: Clin Transl Med. 2021 Sep 26;11(9):e508. doi: 10.1002/ctm2.508 (PMC8473640; doi:10.1002/ctm2.508)
Supplement: Supplementary file 1 — Supporting Information [file CTM2-11-e508-s001.docx]

**Supporting Information**

**Diagnostic and prognostic potential of the oral and gut microbiome for lung adenocarcinoma**

Mi Young Lim^1,#^, Seungpyo Hong^1,#^, Kum Hui Hwang^2^, Eun Jin Lim^2^, Ji-Youn Han^2,*^, Young-Do Nam^1,*^

^1^Research Group of Healthcare, Korea Food Research Institute, Wanju-gun, Jeollabuk-do 55365, Republic of Korea

^2^Center for Lung Cancer, National Cancer Center, Goyang-si, Gyeonggi-do 10408, Republic of Korea

^#^These authors contributed equally to the work.

***Corresponding Authors:**

Young-Do Nam, PhD

Research Group of Healthcare, Korea Food Research Institute, 245 Nongsaengmyeong-ro, Iseo-myeon, Wanju-gun, Jeollabuk-do 55365, Republic of Korea

Email: [youngdo98@kfri.re.kr](mailto:youngdo98@kfri.re.kr)

Tel: +82-63-219-9306

Ji-Youn Han, MD, PhD

Center for Lung Cancer, National Cancer Center, 323 Ilsan-ro, Ilsandong-gu, Goyang-si, Gyeonggi-do 10408, Republic of Korea

Email: [jymama@ncc.re.kr](mailto:jymama@ncc.re.kr)

Tel: +82-31-920-1154

**Supplementary discussion**

We reported the oral and gut microbes differentially abundant in never-smoking lung cancer patients and in healthy controls. The abundance of *Veillonella* was higher in the oral microbiome of the patients. This result is consistent with the previous findings that *Veillonella* was enriched both in the lower airway and saliva of lung cancer patients.^1, 2^ In an *in vitro* study, *Veillonella* products up-regulated the signaling pathways related with carcinogenesis in airway epithelial cells.^2^ *Mogibacterium*, *Butyrivibrio*, and *Variovorax* were more abundant in the oral microbiome of healthy controls. They may contribute to the formation of a healthy microbial community in the mouth, but, to our knowledge, their roles in oral or lung health are mostly unknown, and thus, further studies are required.

In the gut, the abundance of *Faecalibacterium* was reduced in the lung cancer patients. *F. prausnitzii* is considered as a microbe that can modulate systemic immune responses.^3^ A reduction of this genus has been observed in various diseases, such as inflammatory bowel disease, allergic asthma, and colorectal cancer (CRC).^3, 4^ In contrast, the abundance of *Enterococcus* and *Streptococcus* was increased in the gut of lung cancer patients. Their abundance was also enriched in the individuals with CRC.^5^ Gut microbes can influence immune responses at distant body sites as the metabolites produced by them can be delivered to various body parts via the circulatory system.^6^ Therefore, the dysbiosis in the gut may indirectly affect the onset of lung cancer. These examples suggest that the lung cancer-associated microbes reported in this study may have roles in the onset of lung cancer. The causal relationships and mechanisms connecting the gut-lung axis in lung cancer should be elucidated further.

In this study, we revealed a set of novel lung-cancer–associated microbes including *Mogibacterium*, *Butyrivibrio* and *Variovorax*. These discoveries might be caused by the patient’s characteristics. As tobacco smoking has an effect on the oral microbiome,^7^ we only included never-smokers in this study to eliminate the effect of tobacco smoking on the association between the microbiome and lung cancer. Furthermore, we minimized the lung cancer subtype-associated microbiome bias by focusing on lung adenocarcinoma patients. On the contrary, previous studies included patients with various lung cancer subtypes or did not mention the subtypes of patients.^8, 9^ In addition, by investigating both the oral and gut microbiomes, we showed that the oral microbiome may be more strongly associated with lung cancer than the gut microbiome. We also demonstrated that the microbiome profiles can be different by cancer therapy, but the degree of difference was limited. Our results also suggested the prognostic potential of the oral microbiome for lung cancer, at least among patients who have not received cancer treatment.

This study has limitations. Although we demonstrated that the microbial composition of the patients and controls is different, it is unclear whether the microbial alteration affects the onset of lung cancer or the alteration is the result of the patient’s lung cancer status. Further longitudinal studies would be required to uncover this relationship. We suggested that microbiome profiles can be used as biomarkers for the diagnosis of lung cancer. However, as our datasets consisted mostly of late-stage lung cancer patients, further validation with early-stage lung cancer patients is required to test whether the microbiome profile can be used to detect early-stage lung cancer. It may be a limitation that our dataset consisted mostly of female patients (92.3%). However, the predictive model performed well for the independent dataset, which consisted mostly of male patients (92.7%), suggesting that the sex difference may have limited effect on the analysis. Whether the microbiome of lung cancer patients is different depending on sex in the sex-balanced patient dataset needs to be confirmed.

**Methods**

**Study design and sample collection**

We recruited a total of 100 patients who were diagnosed with lung adenocarcinoma and had no lifetime history of smoking from the National Cancer Center Hospital (Goyang, Korea) between April 2018 and August 2019. The cancer histological type was determined according to the 2004 World Health Organization classification of lung tumors. Tumor-node-metastasis (TNM) staging was based on the 8th edition of the lung cancer staging system.^10^

Of the 100 patients enrolled, 91 were included in this study (1 patient was lost at follow-up; 8 failed quality control) (Cancer, n = 91). At the time of microbiome sampling, 39 patients had not received any treatment for lung cancer (Treatment–, n = 39), whereas 52 patients had received one or more lines of chemotherapy or targeted drug therapy for lung cancer (Treatment+, n = 52) since their first diagnosis of lung cancer.

Patients’ fecal samples were collected in OMNIgene-GUT tubes according to the manufacturer's instructions (DNA Genotek, Ottawa, Canada) and were stored at room temperature until use. For collection of saliva samples, subjects were requested to refrain from eating, drinking, and brushing teeth for an hour prior to sample collection. They were asked to rinse their mouths with water and spit 2–5 ml of saliva into a 50 ml tube. Saliva samples were stored at - 80℃ until use.

Control subjects were selected from a previous Korean microbiome study^11, 12^ by selecting never-smoking subjects and then matching them for age and sex to the patients (Control, n = 91). Subjects with a history of various chronic, clinically significant diseases, major gastrointestinal diseases, or cancer, and those who had been administered antibiotics within 3 months before sample collection were not included in the Korean microbiome study.^11, 12^ The entire process from fecal and saliva sampling to 16S rRNA gene sequencing of control samples was performed with the same protocol used for patients’ samples.

For validation of the predictive models, we constructed an independent dataset using the oral and gut microbiome data of 41 Korean non-small cell lung carcinoma patients^13^ and 612 healthy Korean individuals with no smoking history,^11, 12^ which does not include the control subjects used in training the models.

**DNA extraction, 16S rRNA gene sequencing, and sequencing data analysis**

Total DNA from fecal samples was extracted using a QIAamp DNA Stool Mini Kit (Qiagen, Hilden, Germany) with some modification.^14^ Total DNA from saliva samples was extracted using a QIAamp DNA Microbiome Kit (Qiagen). The total DNA extracted from fecal and oral samples was subjected to 16S rRNA V3-V4 library preparation according to the 16S Metagenomic Sequencing Library Preparation Illumina protocol (Part # 15044223 Rev. B, Illumina, San Diego, CA, USA). Sequencing was conducted using an Illumina MiSeq instrument (Illumina). Raw reads of 16S rRNA gene sequencing data were processed using QIIME2 (2019.10 version).^15^ The sequence quality control and feature table construction were performed with the q2-dada2 plugin.^16^ Alpha and beta diversity metrics were calculated using the q2-diversity plugin at an even sampling depth of 10,000 sequences per sample. Taxonomic groups were assigned to the amplicon sequence variants (ASVs) using a pre-trained naïve Bayes classifier against the Greengenes 13.8 99% operational taxonomic units databases^17^ with the q2-feature-classifier plugin.^18^

**Statistical analyses**

Differences in subjects’ characteristics between lung adenocarcinoma patients and healthy controls were evaluated using Student’s t-test or Fisher’s exact test. The Shannon diversity of the microbiome between groups of subjects was calculated using the Wilcoxon rank-sum test or Kruskal-Wallis test. The association of lung cancer with beta diversity was evaluated by permutational multivariate analysis of variance (PERMANOVA) on Bray-Curtis distance using the Adonis function in the vegan R package. Differentially abundant genera between groups of subjects were identified using a Wilcoxon rank-sum test, where the tests were performed on the genera whose prevalence was higher than 0.2. The p-values were adjusted using the Benjamini–Hochberg false discovery rate (FDR) method, and microbial features with q < 0.1 were reported.

**Predictive models for cancer status**

The cancer status for each participant was numerically encoded as 1 if the individual belonged to the non-disease population and as 0 if the individual belonged to the cancer patient population. The relative abundance of bacterial taxa was transformed using the logarithm function with base 10 after adding a pseudo count of 0.00005. The Bayesian Ridge method in Scikit-learn package (Version 0.22.1) was used to predict the cancer status using the transformed microbial abundance values.^19^

A 5-fold cross validation approach was employed to measure the predictive performance. The cross validation itself was repeated five times to reduce the bias caused by the split of datasets into a training and a test set. The false-positive rates and the true positive rates of each test set were used to draw the receiver operating characteristic (ROC) curve and to calculate the average area under the curve (AUC). For the evaluation of the contribution of each bacterial taxon in the predictive models, the coefficients of the Bayesian Ridge model were extracted and normalized by dividing them by the maximum absolute coefficient of the model.

For measurement of the prediction performance in an independent dataset, the Bayesian Ridge models were stored as files. The models were used to predict the cancer status of participants from the independent dataset, and the ROC AUC was measured for each predictive model to measure the performance of the models. The relative abundance data from the independent dataset was used as input of the model after matching the bacterial taxa to those used during training of the model.

**Prognostic analysis**

Lung cancer patients were clustered into two groups based on Bray-Curtis distances using the partition around medoids (PAM) algorithm of the PyClustering software package (v. 0.10.1).^20^ Kaplan-Meier curves were drawn, and the log-rank test was performed with survival and survminer R packages. Predictive models were constructed as described in section 2.4, but with datasets for the two lung cancer patient groups.

**References**

1. Yan X, Yang M, Liu J, et al. Discovery and validation of potential bacterial biomarkers for lung cancer. *Am J Cancer Res* 2015;5:3111-3122.

2. Tsay JJ, Wu BG, Badri MH, et al. Airway microbiota is associated with upregulation of the PI3K pathway in lung cancer. *Am J Respir Crit Care Med* 2018;198:1188-1198. doi:10.1164/rccm.201710-2118OC

3. Ferreira-Halder CV, Faria AVS, Andrade SS. Action and function of Faecalibacterium prausnitzii in health and disease. *Best Pract Res Clin Gastroenterol* 2017;31:643-648. doi:10.1016/j.bpg.2017.09.011

4. Demirci M, Tokman HB, Uysal HK, et al. Reduced Akkermansia muciniphila and Faecalibacterium prausnitzii levels in the gut microbiota of children with allergic asthma. *Allergol Immunopathol (Madr)* 2019;47:365-371. doi:10.1016/j.aller.2018.12.009

5. Cheng Y, Ling Z, Li L. The Intestinal Microbiota and Colorectal Cancer. *Front Immunol* 2020;11:615056. doi:10.3389/fimmu.2020.615056

6. Budden KF, Gellatly SL, Wood DL, et al. Emerging pathogenic links between microbiota and the gut-lung axis. *Nat Rev Microbiol* 2017;15:55-63. doi:10.1038/nrmicro.2016.142

7. Wu J, Peters BA, Dominianni C, et al. Cigarette smoking and the oral microbiome in a large study of American adults. *ISME J* 2016;10:2435-2446. doi:10.1038/ismej.2016.37

8. Yang J, Mu X, Wang Y, et al. Dysbiosis of the Salivary Microbiome Is Associated With Non-smoking Female Lung Cancer and Correlated With Immunocytochemistry Markers. *Front Oncol* 2018;8:520. doi:10.3389/fonc.2018.00520

9. Hosgood HD, Cai Q, Hua X, et al. Variation in oral microbiome is associated with future risk of lung cancer among never-smokers. *Thorax* 2021;76:256-263. doi:10.1136/thoraxjnl-2020-215542

10. Detterbeck FC, Boffa DJ, Kim AW, Tanoue LT. The Eighth Edition Lung Cancer Stage Classification. *Chest* 2017;151:193-203. doi:10.1016/j.chest.2016.10.010

11. Lim MY, Hong S, Bang SJ, et al. Gut microbiome structure and association with host factors in a Korean population. *mSystems* Under review;

12. Lim MY, Hong S, Kim JH, Nam YD. Oral microbiome features of demographic groups in a Korean population. *Manuscript in preparation*

13. Lim MY, Hong S, Han JY, Nam YD. Microbial biomarkers for predicting efficacy of immunotherapy in lung cancer. *Manuscript in preparation*

14. Lim MY, Song EJ, Kim SH, Lee J, Nam YD. Comparison of DNA extraction methods for human gut microbial community profiling. *Syst Appl Microbiol* 2018;41:151-157. doi:10.1016/j.syapm.2017.11.008

15. Bolyen E, Rideout JR, Dillon MR, et al. Reproducible, interactive, scalable and extensible microbiome data science using QIIME 2. *Nat Biotechnol* 2019;37:852-857. doi:10.1038/s41587-019-0209-9

16. Callahan BJ, McMurdie PJ, Rosen MJ, Han AW, Johnson AJ, Holmes SP. DADA2: High-resolution sample inference from Illumina amplicon data. *Nat Methods* 2016;13:581-583. doi:10.1038/nmeth.3869

17. DeSantis TZ, Hugenholtz P, Larsen N, et al. Greengenes, a chimera-checked 16S rRNA gene database and workbench compatible with ARB. *Appl Environ Microbiol* 2006;72:5069-5072. doi:10.1128/AEM.03006-05

18. Bokulich NA, Kaehler BD, Rideout JR, et al. Optimizing taxonomic classification of marker-gene amplicon sequences with QIIME 2's q2-feature-classifier plugin. *Microbiome* 2018;6:90. doi:10.1186/s40168-018-0470-z

19. Pedregosa F, Varoquaux G, Gramfort A, et al. Scikit-learn: Machine learning in Python. *J Mach Learn Res* 2011;12:2825-2830.

20. Novikov AV. PyClustering: Data Mining Library. *Journal of Open Source Software* 2019;4:1230. doi:10.21105/joss.01230

**Table S1. Characteristics of the study subjects**

|  | Training dataset | | | Independent test dataset | | |
| --- | --- | --- | --- | --- | --- | --- |
| Characteristic | Healthy controls  (n = 91) | Lung cancer patients  (n = 91) | p-value | Healthy controls  (n = 612) | Lung cancer patients  (n =41) | p-value |
| Age (years) (mean ± SD) | 63.5 ± 9.84 | 63.5 ± 9.82 | 0.988^†^ | 53.95 ± 15.46 | 62.63 ± 9.45 | < 0.001^†^ |
| Sex, n (%) |  |  | 0.767^‡^ |  |  | < 0.001^‡^ |
| Male | 5 (5.5) | 7 (7.7) |  | 43 (7.0) | 38 (92.7) |  |
| Female | 86 (94.5) | 84 (92.3) |  | 569 (93.0) | 3 (7.3) |  |
| Tumor stage, n (%) |  |  |  |  |  |  |
| I |  | 1 (1.1) |  |  | 0 (0.0) |  |
| II |  | 1 (1.1) |  |  | 0 (0.0) |  |
| III |  | 8 (8.8) |  |  | 2 (4.9) |  |
| IV |  | 81 (89.0) |  |  | 39 (95.1) |  |
| Mutations, n (%) |  |  |  |  |  |  |
| EGFR |  | 59 (64.8) |  |  | 4 (9.8) |  |
| ALK |  | 10 (11.0) |  |  | 0 (0.0) |  |
| KRAS |  | 6 (6.6) |  |  | 6 (14.6) |  |
| ERBB2 |  | 2 (2.2) |  |  | 0 (0.0) |  |
| BRAF |  | 1 (1.1) |  |  | 0 (0.0) |  |
| ROS1 |  | 1 (1.1) |  |  | 0 (0.0) |  |
| Unknown |  | 12 (13.2) |  |  | 31 (75.6) |  |
| Line of treatment, n (%) |  |  |  |  |  |  |
| 0 |  | 39 (42.9) |  |  | 4 (9.8) |  |
| 1 |  | 22 (24.2) |  |  | 22 (53.7) |  |
| 2 |  | 7 (7.7) |  |  | 12 (29.3) |  |
| ≥3 |  | 23 (25.3) |  |  | 3 (7.3) |  |

^†^Student’s t-test; ^‡^Fisher’s exact test

**Supplementary Figures**


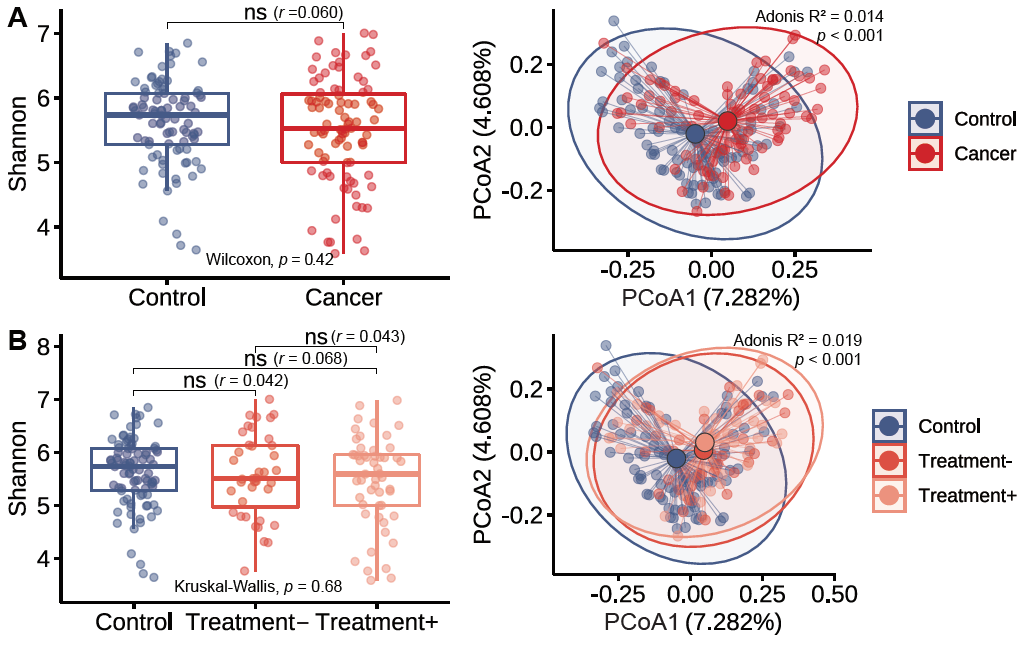


**Figure S1. Gut microbiome diversity in healthy controls and lung cancer patients.** Shannon’s diversity index (left) and principal coordinate analysis plots (right) based on the Bray-Curtis distance. Samples were divided into healthy controls and lung cancer patient groups (A), or healthy controls, Treatment–, and Treatment+ groups (B). ns, not significant; Wilcoxon rank-sum test. *r* means Wilcoxon effect size.


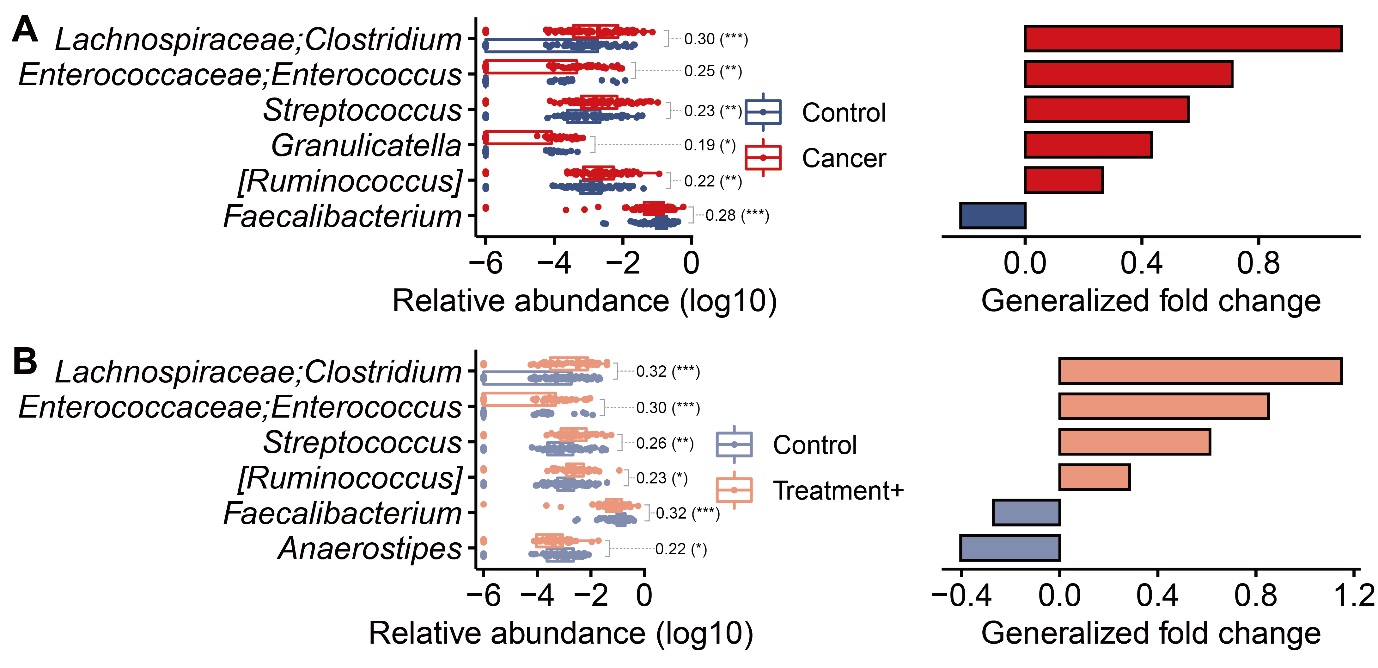


**Figure S2. Gut bacteria genera that were differentially represented in the lung cancer patients and control groups.** Log_10_-transformed relative abundance (left) and generalized fold change (right) of differentially abundant genera among the different groups of subjects. Differentially abundant genera in healthy controls versus (A) all lung cancer patients or (B) Treatment+ group were identified using a Wilcoxon rank-sum test (q < 0.1). * q < 0.1; ** q < 0.05; *** q < 0.01; The numbers between groups in the boxplot indicate the Wilcoxon effect size.


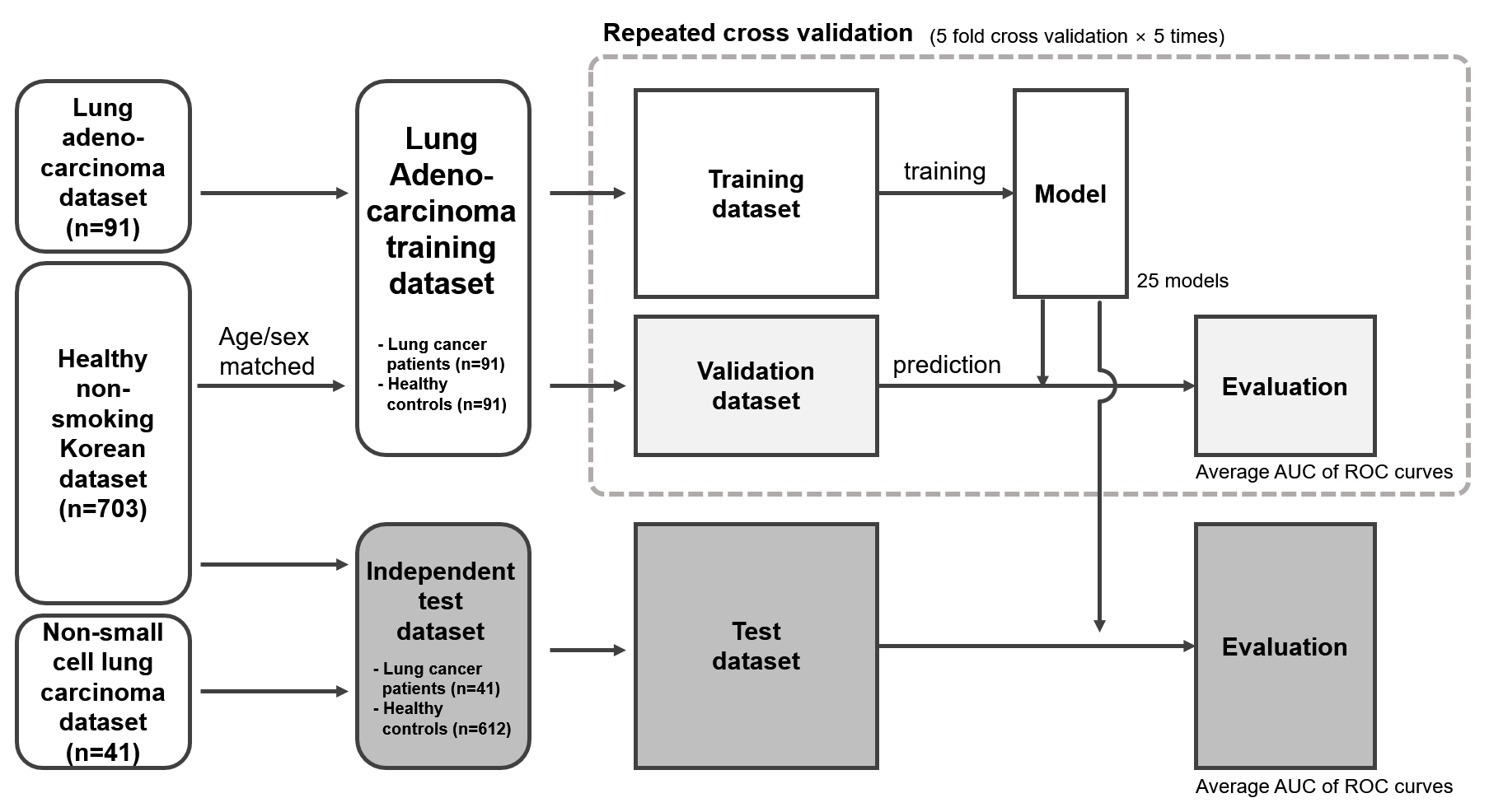


**Figure S3. Machine-learning workflow.** The lung adenocarcinoma dataset and the age and sex-matched healthy non-smoking Korean dataset were compiled into a dataset to train the lung adenocarcinoma prediction model. The remaining healthy non-smoking Korean dataset and the dataset from the non-small cell lung carcinoma dataset, which is independent of the training dataset, were combined into an independent test dataset. The lung adenocarcinoma training dataset was randomly separated into training and validation datasets to train and evaluate the prediction performance of the model. For the training and validation, a five-cross validation technique was employed, and the cross-validation was repeated five times. The trained models were applied to the independent test dataset to measure their predictive performance on the independent dataset.


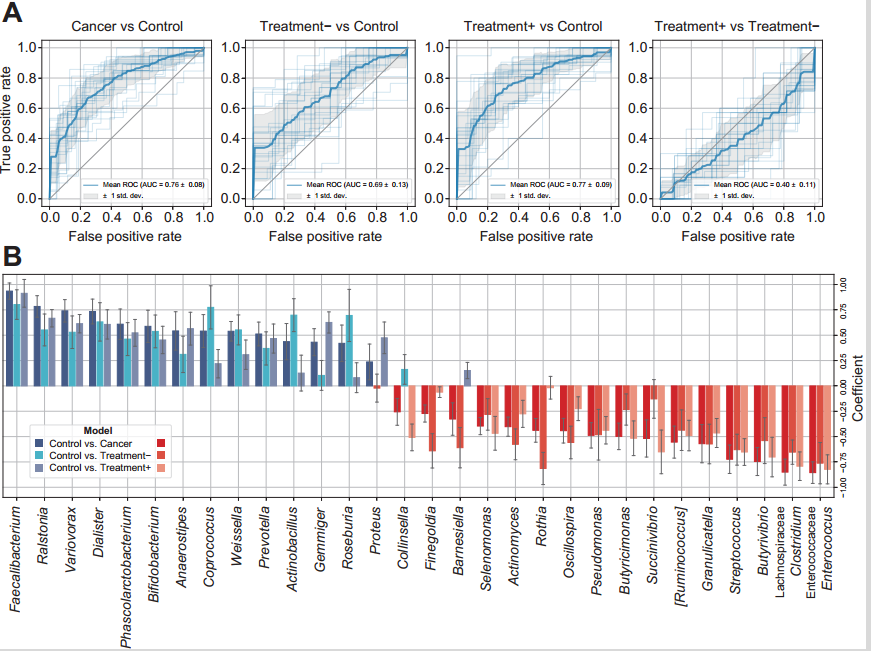


**Figure S4. Lung cancer prediction from gut microbiome signatures.** (A) Receiver operating characteristic (ROC) curve of the predictive models that distinguish total cancer patients, Treatment+ group, Treatment– group, and control group from each other. The ROC curves of the test sets are plotted with semi-transparent lines and the average curve is plotted with a solid line. The area representing one standard deviation from the average is colored in gray. The area under curve (AUC) is shown in the islet. (B) Normalized coefficients of the predictive models (Bayesian Ridge). The genera for which the coefficients belonged to the highest and lowest 5^th^ percentiles in the predictive model of Cancer vs Control are displayed. The higher abundance of a microbe with a positive coefficient increases the chance of predicting a healthy control.
